# Prevalence and risk factors of sleep disturbances in a large HIV-infected adult population

**DOI:** 10.7448/IAS.17.4.19576

**Published:** 2014-11-02

**Authors:** Clotilde Allavena, Thomas Guimard, Eric Billaud, Sylvie de la Tullaye, Véronique Reliquet, Solène Pineau, Hervé Hüe, Christelle Supiot, Jean Marie Chennebault, Christophe Michau, Hikombo Hitoto, Rémi Vatan, François Raffi

**Affiliations:** 1 Infectious Diseases, CHU Hôtel-Dieu, Nantes, France; 2 Infectious Diseases, CHD Vendée, La Roche sur Yon, France; 3 Infectious Diseases, CHU Hôtel-Dieu, COREVIH Pays de la Loire, Nantes, France; 4 CHU, Explorations Fonctionnelles, Nantes, France; 5 Infectious Diseases, CHU, Angers, France; 6 CH, Médecine, St Nazaire, France; 7 Infectious Diseases, CH, Le Mans, France; 8 Médecine Interne, CH, Laval, France

## Abstract

**Introduction:**

Sleep disturbances are frequently reported in HIV-infected patients but there is a lack of large studies on prevalence and risk factors, particularly in the context of current improved immuno-clinical status and use of the newest antiretrovirals (ARV).

**Method:**

Cross-sectional study to evaluate the prevalence and factors associated with sleep disturbance in adult HIV-infected patients in six French centres of the region “Pays de la Loire”. Patients filled a self-administered questionnaire on their health behaviour, sleep attitudes (Pittsburgh Sleep Quality Index PSQI), quality of life (WHO QOL HIV BREF questionnaire) and depression (Beck depression Inventory (BDI)-II questionnaire). Socio-demographic and immunovirologic data, medical history, ARVs were collected.

**Results:**

From November 2012 to May 2013, 1354 consecutive non-selected patients were enrolled. Patients’ characteristics were: 73.5% male, median age 47 years, active employment 56.7%, France-native 83% and Africa-native 14.7%, CDC stage C 21%, hepatitis co-infection 13%, lipodystrophy 11.8%, dyslipidemia 20%, high BP 15.1%, diabetes 3%, tobacco smokers 39%, marijuana and cocaine users, 11.7% and 1.7% respectively, and excessive alcohol drinkers 9%. Median (med) duration of HIV infection was 12.4 years, med CD4 count was 604/mm^3^; 94% of Patients were on ARVs, 87% had undetectable viral load. Median sleeping time was 7 hours. Sleep disturbances (defined as PSQI score >5) were observed in 47% of the patients, more frequently in female (56.4%) than in male (43.9%) (p<0.05) and moderate to serious depressive symptoms (BDI score>19) in 19.7% of the patients. In multivariate analysis, factors associated with sleep disturbances (p<0.05) were depression (odds ratio [OR] 4.6; 95% confidence interval [CI] 3.2–6.8), male gender (OR 0.7; CI 0.5–0.9), active employment (OR 0.7; CI 0.5–0.9), living single (OR 1.5; CI 1.2–2.0), tobacco-smoking (OR 1.3; CI 1.0–1.8), duration of HIV infection (>10 vs. <10 y.) (OR 1.5; CI 1.1–2.0), ARV regimen containing nevirapine (OR 0.7; CI 0.5–0.9) or efavirenz (OR 0.5; CI 0.3–0.7).

**Conclusions:**

Prevalence of sleep disturbances is high in this HIV population and roughly similar to the French population. Associated factors are rather related to social and psychological status than HIV infection. Depression is frequent and should be taken in care to improve sleep quality.

